# Green Synthesis via *Eucalyptus globulus* L. Extract of Ag-TiO_2_ Catalyst: Antimicrobial Activity Evaluation toward Water Disinfection Process

**DOI:** 10.3390/nano12111944

**Published:** 2022-06-06

**Authors:** Jacqueline Torres-Limiñana, Ana A. Feregrino-Pérez, Marina Vega-González, Luis Escobar-Alarcón, José Antonio Cervantes-Chávez, Karen Esquivel

**Affiliations:** 1 División de Investigación y Posgrado, Facultad de Ingeniería, Universidad Autónoma de Querétaro, Cerro de las Campanas, Santiago de Queretaro 76010, Mexico; jacqueline.321@hotmail.com (J.T.-L.); feregrino.angge@hotmail.com (A.A.F.-P.); 2 Centro de Geociencias, Universidad Nacional Autónoma de México, Campus Juriquilla. Blvd. Juriquilla, 3001, Santiago de Queretaro 76230, Mexico; mvega@geociencias.unam.mx; 3 Departamento de Física, ININ, Carr. México-Toluca, La Marquesa, Ocoyoacac 52750, Mexico; luis.escobar@inin.gob.mx; 4 Facultad de Ciencias Naturales, Universidad Autónoma de Querétaro, Carr. Chichimequillas-Anillo Vial Fray Junípero Serra, Km 8, Santiago de Queretaro 76000, Mexico; jose.antonio.cervantes@uaq.mx

**Keywords:** antimicrobial activity, catalyst, *Eucalyptus globulus* L., green synthesis, silver nanoparticles, titanium dioxide

## Abstract

The problem of water pollution by persistent substances and microorganisms requires solutions that materials such as silver-modified titanium dioxide can provide due to their excellent photocatalytic and antimicrobial properties. However, the synthesis methods conventionally used to obtain these materials involve toxic chemical reagents such as sodium borohydride (NaBH_4_). The search for alternative synthesis methods that use environmentally friendly substances, such as the biosynthesis method, was evaluated. Silver-titanium dioxide (Ag-TiO_2_) was synthesized by a *Eucalyptus globulus* L. extract as a reductive agent through sol-gel and microwave-assisted sol-gel processes. Four different solvents were tested to extract secondary metabolites to determine their roles in reducing silver nanoparticles. Titanium dioxide nanoparticles with sizes from 11 to 14 nm were obtained in the anatase phase, and no narrowing of the bandgap was observed (3.1–3.2 eV) for the Ag-TiO_2_ materials compared with the pure TiO_2_. Interestingly, the bacterial inhibition values were close to 100%, suggesting an effective antimicrobial mechanism related to the properties of silver. Finally, by the physicochemical characterization of the materials and their antimicrobial properties, it was possible to obtain a suitable biosynthesized Ag-TiO_2_ material as a green option for water disinfection that may be compared to the conventional methods.

## 1. Introduction

The conventional synthesis methods of metallic and metallic oxide nanomaterials (NMs) involve the use of hazardous reagents, such as sodium borohydride (NaBH_4_), which is why several studies propose the use of “green synthesis” as a clean and safe synthesis process which is friendly to the environment [1]. Among the emerging synthesis proposals, different biomass sources are used as reducing agents, such as plant, fungi, and microorganism extracts, to obtain nanostructured materials based on metallic compounds. Although the synthesis of nanoparticles (NPs) mediated by plant extracts is relatively easy, some aspects have been challenging to understand. One of these aspects involves understanding which biomolecules present in the extracts are responsible for reducing the metal ions of the precursor and which biomolecules act as stabilizing agents of the nanoparticles [2,3].

Extracts of different species of Eucalyptus have been studied in the synthesis of nanoparticles, possibly due to the high presence of Eucalyptus worldwide [4]. Furthermore, a few studies of the composition of the extracts of different Eucalyptus species were focused on the search for phenolic compounds as the principal reduction agents in the metallic NP synthesis [5,6]. Nevertheless, the proper mechanism of how the plant extracts work in the NP production is still under study. A deeper analysis of the extract composition must be conducted for each plant species used [7,8,9]. Once the nanomaterial was obtained via green synthesis, it was proved that these kinds of NMs can be applied in diverse areas as antimicrobial agents or even as photocatalysts for wastewater treatment [10,11,12,13,14,15].

Among the well-known and used photocatalysts for wastewater treatment is titanium dioxide (TiO_2_) in its anatase phase or anatase-rutile mix phase [16,17,18]. TiO_2_ is a semiconductor, on which, once it is activated under UV light, a surface phenomenon occurs, producing the charge separation (e^−^/h^+^) to conduce the redox reactions and obtain hydroxyl radicals (·OH), which help the mineralization process of the non-biodegradable pollutants and the disinfection process in wastewaters [19,20].

The wastewater treatment by photocatalysis has been intensely studied in the past decades [21], revealing new forms of synthesis methods for the catalysts [22] and ways to to remove persistent, emergent, and complex pollutants, such as aromatic compounds [23], dyes [24], endocrine disrupters [25], personal care products [26], and pharmaceuticals [27,28]. Even with the amount of knowledge and information regarding this topic, more studies are conducted to achieve a scaled-up application, and more studies show that TiO_2_ can be modified with silver NPs to synergize antimicrobial activity, reducing the bandgap energy (3.2 eV) and activating the photocatalyst with visible light [29,30,31,32,33].

The Ag^0^ NP and TiO_2_ synthesis by conventional methods has been widely reported and includes chemical reduction [34], sol–gel [35,36], microwave-coupled sol–gel [37,38], sonochemistry-coupled sol–gel [38,39], PLD [40,41], and sputtering [42,43], to mention just the most representative. Moreover, the green synthesis of both materials in a separate way has been widely studied with different plants, fungi, and microorganism extracts [10,12,44,45,46,47,48,49,50,51,52,53,54,55,56,57]. Nevertheless, only a few works have combined the green synthesis for metallic silver, TiO_2_, or Ag-TiO_2_ materials with *Eucalyptus globulus* L. plant extract. Balamurugan et al. (2017) reported a single-step eco-friendly, energy-efficient, and economically scalable green method to synthesize silver nanoparticles using *Eucalyptus globulus* L. leaf extract as a reducing and capping agent. The mean sizes of the prepared silver nanoparticles ranged from 30 to 36 nm [5]. Balaji et al. (2021) reported the synthesis and characterization of nano-titania (TiO_2_), using *Eucalyptus globulus* L. leaf aqueous extract. The NPs exhibited a pure anatase phase structure with an average particle size of 12 nm [58].

For the material Ag-TiO_2_ obtained by green synthesis, no reports were found using *Eucalyptus globulus* L. extract as a reducing agent. Hariharan et al. (2020) reported Ag@TiO_2_ NPs using *Aloe vera* gel as a capping and reducing agent with excellent photocatalytic activity [59], and Rajkumar et al. (2022) reported Ag-doped TiO_2_ nanoparticles synthesized using grapefruit extract as a solvent/reducing agent [60]. According to the presented information and the previous results obtained by our research group [61,62,63], the proposal is to synthesize Ag-TiO_2_ NMs with a green approach, using *Eucalyptus globulus* L. extract by the microwave-coupled sol–gel method. Moreover, the aim is to evaluate its antimicrobial activity towards the disinfection process of biologically treated wastewaters and the photocatalytic processes of non-biodegradable pollutants. This work aims to prove that by using *Eucalyptus globulus* L. extract it is possible to obtain silver NPs. Coupled with the well-known sol-gel synthesis method, it should be possible to generate Ag-TiO_2_ catalysts with characteristics that are improved or similar to those synthesized by conventional and toxic reagents such as NaBH_4_.

## 2. Materials and Methods

### 2.1. Eucalyptus globulus L. Extract Preparation and Secondary Metabolite Quantification

*Eucalyptus globulus* L. leaves were collected from Quiroga, Michoacán (19°40′ N latitude and 101°32′ W longitude) and from Salvatierra, Guanajuato (latitude: 20.2081, longitude: −100.872, 20°12′29″ N, 100°52′19″ W); both sites are located in Mexico. All parts of the plant (leaves, branches, and fruits) were first washed with distilled water to remove dust particles and then were dried in a Dynamica Air Performance convection oven at 35 °C until their weight was constant.

Subsequently, the branches and fruits were removed, only the leaves were crushed in an industrial blender with dry ice to obtain a powder, which was kept at −81 °C in the dark. The extraction was conducted in a powder/liquid ratio of 1/10 (*w*/*v*) in a Branson 510 ultrasonic bath at 50 °C for 30 min, using different solvent mixtures: ethanol ((Mexico City, CDMx, Mexico) JT Baker) (EtOH), methanol (JT Baker, Phillipsburg, NJ, USA) (MeOH), 50/50 ethanol/water (EtOH/H_2_O), and 50/50 methanol/water (MeOH/H_2_O) (*v*/*v*). The solid part of the extract was separated by filtration, maintaining dark conditions to avoid the photodegradation of the extract. Furthermore, the liquid part was centrifugated for 20 min at 8500 rpm and kept in the dark at −4 °C for the following secondary metabolite determinations and silver NP reduction experiments.

The extracts’ total phenolic and flavonoid contents were determined according to the Folin–Ciocalteu spectrophotometric method, modified for a 64-well microplate. The total phenol content results were expressed as the equivalent mg of gallic acid per gram of fresh sample, and rutin hydrate (flavonoid) was expressed as the equivalent mg of rutin per gram of fresh sample.

The extracts’ antioxidant activity was evaluated by the 2,2-diphenyl-1-picrylhydrazyl (DPPH) radical method, the results of which were expressed as the percentage of DPPH discoloration (% radical inhibition), also known as percentage inhibition (IHB), and by the ABTS assay. All the spectrophotometric measurements were obtained by a Thermo Scientific Multiskan Go spectrophotometer. Statistical analysis was performed using JMP (JMP-statistical trial 15). The ANOVA test used a Tukey pairwise comparison to determine significant statistical differences. A Games–Howell pairwise comparison was made for the data not showing equal variances, and the significance value was set to α ≤ 0.05.

### 2.2. Catalyst’s Synthesis and Characterization

The pure silver NPs were synthesized to compare the ones obtained via green synthesis and those obtained by conventional synthesis. The silver nanoparticles (Ag NPs) were obtained by reducing 1mM AgNO_3_ (JT Baker) and plant extract in a 1:10 volumetric ratio of extract:AgNO_3_ and allowed to react for 4 h under stirring at 40 °C. After that, for the next 20 h they were kept in the dark at room temperature and still under stirring. Subsequently, the Ag NPs were washed and centrifuged at 8500 rpm for 20 min. Finally, they were resuspended in distilled water for characterization and dried when used in the antimicrobial tests.

Silver nitrate was used as a precursor for the conventional synthesis and NaBH_4_ (JT Baker) 0.01M as a reducing agent in a 1:2 NaBH_4_:AgNO_3_ volume ratio. The time for reaction was 75 min under stirring and in the dark. For both synthesis methods, the characterization of the Ag NPs was carried out using the UV–Vis technique to evaluate the formation of the nanoparticles in a UV Detective Plus XB-10 spectrophotometer in a wavelength range from 200 to 800 nm.

The TiO_2_ catalysts were prepared by the sol-gel and microwave-coupled sol-gel methods. Titanium isopropoxide (TTIP, Toluca, Edo. Mex., Mexico, Sigma Aldrich, 97%) was dissolved in isopropanol (JT Baker 99%). The solution was stirred for 20 min under a nitrogen atmosphere, and the hydrolysis process was performed by adding water to the precursor/solvent solution, and this new solution was then stirred for 1 h. The molar ratio was 0.04:1.3:160:0.0025 (TTIP: Isopropanol: Water: AgNO_3_). For the Ag-modified TiO_2_, the precursor AgNO_3_ (JT Baker) was used; it was added by dissolving it into the water used for the hydrolysis. For the conventional synthesis, the NaBH_4_ agent was added in this step. For the green synthesis, the *Eucalyptus globulus* L. extract was added.

The obtained product was dried at room temperature and then calcined at 450 °C for 30 min to promote the anatase crystal phase. For this synthesis, the materials were identified as SG. The samples synthesized by the microwave-coupled sol-gel process, in the suspension before the filtration process, were transferred into a Teflon vessel and placed on a microwave reaction system (Flexiwave Milestone). The process was carried out at a temperature of 215 °C for 30 min. Once the product was obtained, it was filtered, dried, and calcined at 450 °C for 30 min. For this synthesis, the materials were identified as MW.

Morphology analyses were carried out using a JEOL (JEOL, Peabody, MA, USA) JSM-6010 LV scanning electron microscope operating at 15 keV and a JEOL JEM 2000FX transmission electron microscope. The crystallinity was determined by X-ray Diffraction analysis (XRD), using a Bruker D8 advanced diffractometer equipped with a Cu anode to generate Cu Kα radiation (λ = 1.5406 Å), with angles of 10 < 2*θ* < 80°, using a step size of 0.01°. Raman measurements were performed using a micro-Raman LabRam 800 system, equipped with a confocal microscope Olympus BX40 and a 100Χ objective; the samples were excited using the second harmonic of an Nd:YAG laser (532 nm). Diffuse reflectance measurements (DRS) were conducted in a Perkin Elmer Lambda 35 UV–Vis spectrophotometer to obtain the bandgap values through the Kubelka–Munk function [64]. The XPS spectra were acquired in the low- and high-resolution regimes with a K-alpha Thermo Scientific (Waltham, MA, USA) XPS spectrometer. The adventitious carbon peak at 284.8 eV was used as the internal standard to compensate for sample charging. All measurements were made in an ultra-high vacuum (UHV) chamber at pressures between 5 × 10^−9^ and 2 × 10^−8^ Torr.

### 2.3. Antimicrobial Evaluation by Direct Contact

The antimicrobial activity of the materials was evaluated using *Escherichia coli* as Gram-negative bacteria and *Staphylococcus aureus* as Gram-positive bacteria. One milliliter of bacteria was inoculated in a Luria–Bertani (LB) medium and adjusted at 600 nm to an optical density of 0.07. The material was dispersed in distilled water at a concentration of 100 μg/μL and dispersed for 30 min in an ultrasonic bath (Branson 2510) and sterilized before use. Two hundred microliters of the material’s solution was taken and placed with 300 μL of LB medium in a new and sterile microtube maintaining a ratio of 4:10 (material solution: LB medium). The volume ratios for the incubation of the bacteria with the treatment are indicated in Table 1. The amounts of medium, bacteria, and material were set to have a volume of 450 μL, and aliquots of 150 μL were prepared in 1.5 mL tubes; the remaining 150 μL was discarded.

The samples were incubated at 37 °C in the dark for 24 h. The final concentration of the material used was 13.33 μg/μL per treatment. After the incubation time, one mL of tenfold serial dilution was prepared using physiological solution (=0.85% NaCl). The last dilution was spread in duplicate Petri dishes with LB agar and incubated at 37 °C for 24 h. Afterwards, the colony-forming units (UFC) were scored.

## 3. Results

### 3.1. Plant Extract Characterization

#### 3.1.1. UV–Vis and IR Spectroscopies

**Figure 1:**
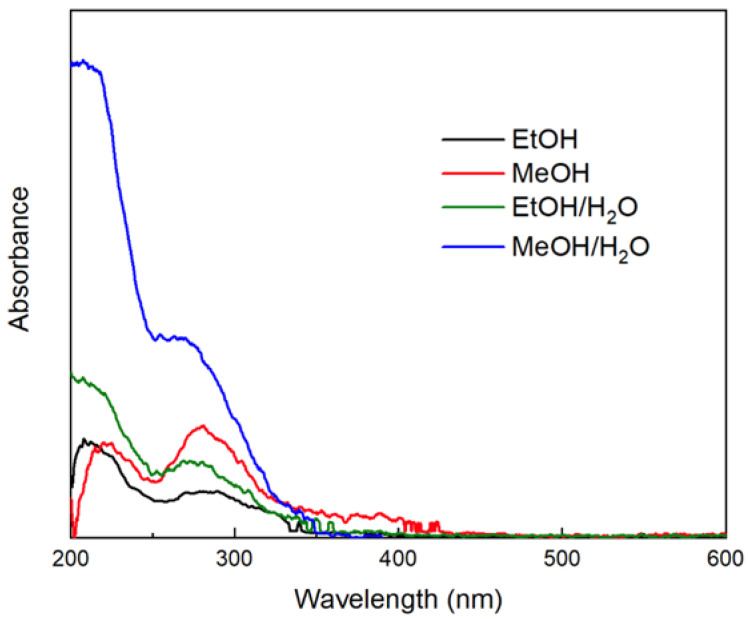
Plant extract absorbance spectra from different types and mixtures of solvents.

Once the *Eucalyptus globulus* L. extract was obtained, UV–Vis spectroscopy was carried out. In Figure 1, it is possible to observe an absorbance signal between 200 and 250 nm, which corresponds to the π–π* transition of conjugated C=C bonds, and the second absorbance signal between 260 and 300v nm corresponds to the π–π* transition related to C=O bonds [65]. The increase in the absorbance of the UV–Vis spectrum is related to the concentration of the components in the solution. However, it is not necessary for the effectiveness of the extraction process. The MeOH/water extract indicates that the extraction process is greater than the rest of the solvents as the absorbance increases, as shown by the blue line in Figure 1 [66].

**Figure 2:**
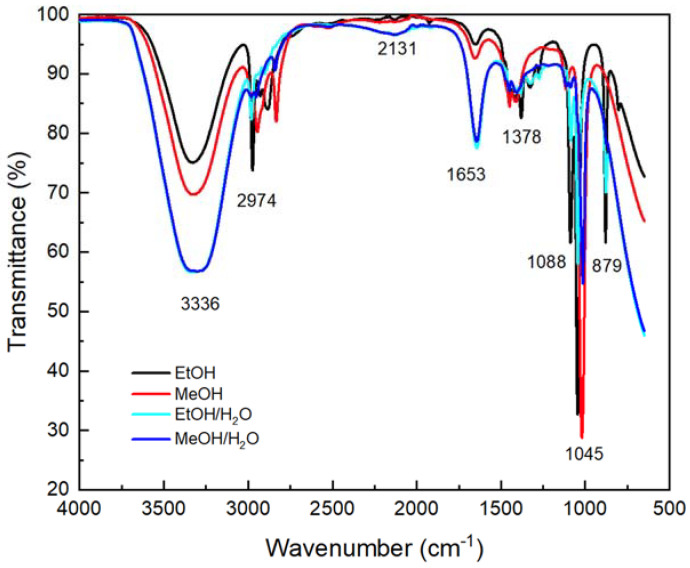
Plant extract FT-IR spectra from different types of solvents.

In Figure 2, the various signals shown in the different solvent–plant extract FT-IR analyses are related to the different organic compounds found in the samples, specifically those within their structures, O–H bonds, carboxyl groups, and C=O bonds. The signals are in good agreement with those present in the *Eucalyptus globulus* L. extract and its active compounds, including ellagic acid, eucalyptone, and macrocarpal A and E [65,67,68].

The presence of water as solvent produces changes in the intensity of some signals, which coincide with those associated with the 3336 cm^−1^ of O–H bond stretching; 2131 cm^−1^, the earwig-like flexing and a broad near-IR release band; and 1653 cm^−1^, associated with the scissor bending of the O–H–O bond [69]. The signal at 2974 cm^−1^ corresponds to C–H bond vibration; 1378 cm^−1^ corresponds to carboxyl group (–COOH) absorption; 1088 and 1045 cm^−1^ correspond to the C–N bond (aliphatic amines of phenolic compounds) absorption; and 879 cm^−1^ to out-of-plane aromatic C–H deformation. All the observed bands support the presence of phytochemical constituents with phenols, amines, carboxyl, and carbonyl functional groups [70,71].

#### 3.1.2. Secondary Metabolites Evaluation

The plant extract with the highest content of phenolic compounds was the one where ethanol works as a solvent, followed by ethanol/water, methanol, and methanol/water. A significant decrease is observed when methanol and methanol combined with water are used (Table 2). This suggests that the water affects the capacity to extract these compounds, in comparison with the pure solvent, and that the ethanol is more akin to the extraction of the phenolic compounds present in eucalyptus [72].

Gullón et al. (2017) report a higher content of flavonoids and phenols when a mixture of two solvents is used (with longer extraction times) [73], contrary to what was observed in the results of this work for phenols. This could be because the proportions of alcohol used in the alcohol/water mixtures are high. It is reported that after a point of maximum efficiency, a more significant amount of alcohol decreases the content of flavonoids and phenols due to the short extraction time used [74]. A higher value of TEAC (Trolox Equivalent Antioxidant Capacity) corresponds to a higher capacity to eliminate the DPPH radical [75]. The different types of bioactive compounds extracted depend on the variations between solvents. DPPH and ABTS are based on the ability of the antioxidant compound (*Eucalyptus globulus* L. extract) to neutralize free radicals (2,2-diphenyl-1-picrylhydrazyl and 2,2′-azino-bis-(3-ethyl benzothiazoline-6-sulfonic acid) [73].

Methanol, ethanol, and methanol/water presented higher antioxidant activity by ABTS, followed by ethanol/water, as shown in Table 2. There is no statistical difference in the ABTS antioxidant activity between the two pure alcohols and the methanol/water mixture. However, for the ethanol/water extract, the inhibition percentage of ABTS decreases. It may be related to the solvent’s affinity over the free radical trapping ability. Moreover, it has been observed that some antagonistic effects between compounds reduce the anti-radical effect [76]. Compared with the DPPH antioxidant activity, the highest inhibition percentages were obtained with the alcohols/water mixtures with no statistical difference, followed by the ethanol extract, and finally for the methanol extract. This effect could also be related to the solvent’s affinity over the free radical trapping ability. It is possible to mention that the present compounds in the *Eucalyptus globulus* L. extracts confer antioxidant activity, which will be helpful as a reduction agent for metallic NP biosynthesis.

### 3.2. Physicochemical Characterization of Ag^0^, TiO_2,_ and Ag-TiO_2_ Materials

#### 3.2.1. UV-Visible Spectroscopy

**Figure 3:**
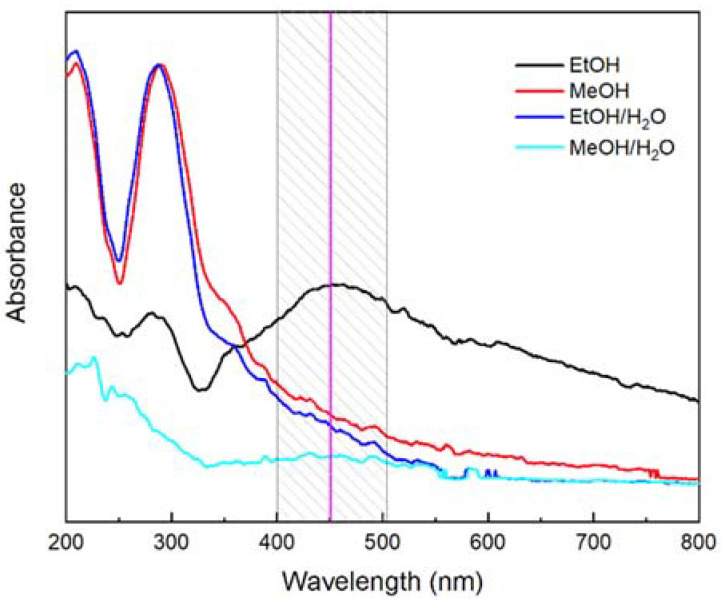
Ag NPs UV–Visible spectra from plant extract obtained by different types of solvents.

An indication of the formation of silver nanoparticles was when the color changed from a light green color with a transparent appearance to a darker green after 4 h of stirring at 40 °C in darkness. The reaction was allowed to complete for the next 18 h. In Figure 3, the UV–Vis spectra of the synthesis products carried out with the different eucalyptus extracts are presented. As is seen, only the spectrum of the sample prepared with ethyl alcohol (EtOH) shows an absorbance band around 453 nm, which is attributed to the surface plasmon of silver [77,78]. This result indicates that the only extract capable of reducing the silver nitrate was the one prepared with EtOH.

The signals at wavelengths lower than 400 nm are attributed to traces of the extract that were not eliminated despite the purification and the washing steps to which the nanoparticles were subjected prior to their characterization.

The ethanol extracted secondary metabolites with the ability to reduce silver nitrate. Nevertheless, from the evaluation of the secondary metabolites, it was shown that the extracts obtained with ethanol and ethanol/water were the most efficient in the extraction of phenols and had a good content of flavonoids, along with the DPPH antioxidant activity, suggesting that these metabolites were not the only ones involved in the biosynthesis process. Other types of compounds, such as tannins, monoterpenes, and polyphenols, may have contributed to the formation and stabilization of the nanoparticles [79,80,81].

#### 3.2.2. SEM and TEM Characterization

**Figure 4:**
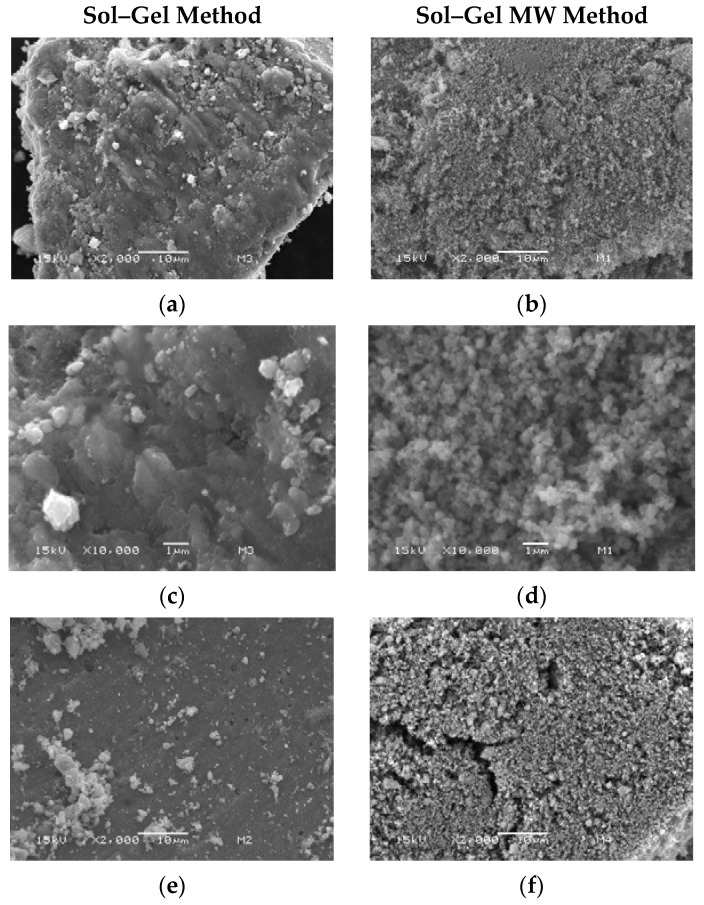

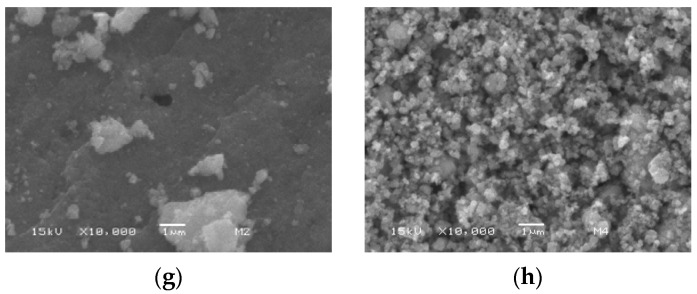
SEM micrographs of (**a**) NaBH_4_/Ag-TiO_2_ SG 2000×, (**b**) NaBH_4_/Ag-TiO_2_ MW 2000×, (**c**) NaBH_4_/Ag-TiO_2_ SG 10,000×, (**d**) NaBH_4_/Ag-TiO_2_ MW 10,000×, (**e**) plant extract/Ag-TiO_2_ SG 2000×, (**f**) plant extract/Ag-TiO_2_ MW 2000×, (**g**) plant extract/Ag-TiO_2_ SG 10,000×, (**h**) plant extract/Ag-TiO_2_ MW 10,000×.

For the sol–gel method, both reducing reagents, NaBH_4_ and the plant extract, did not differ in morphology for the Ag-TiO_2_ materials (Figure 4a,c,e,g). It can be seen as a non-defined, non-uniform surface with no homogeneity of sizes. In the case of the microwave-assisted sol–gel method, an amorphous and non-homogeneous morphology is observed. Moreover, there is no difference when the NaBH_4_ or the plant extract reduces the silver ions (Figure 4b,d,f,h). As expected, the difference observed in the SEM micrographs is due to the use of an external energy source, such as the MW [38,61,63]. A spongy morphology is observed compared to the sol–gel synthesis method, which could lead to smaller particle size and, in the case of the eucalyptus extract, could stabilize the particles.

**Figure 5:**
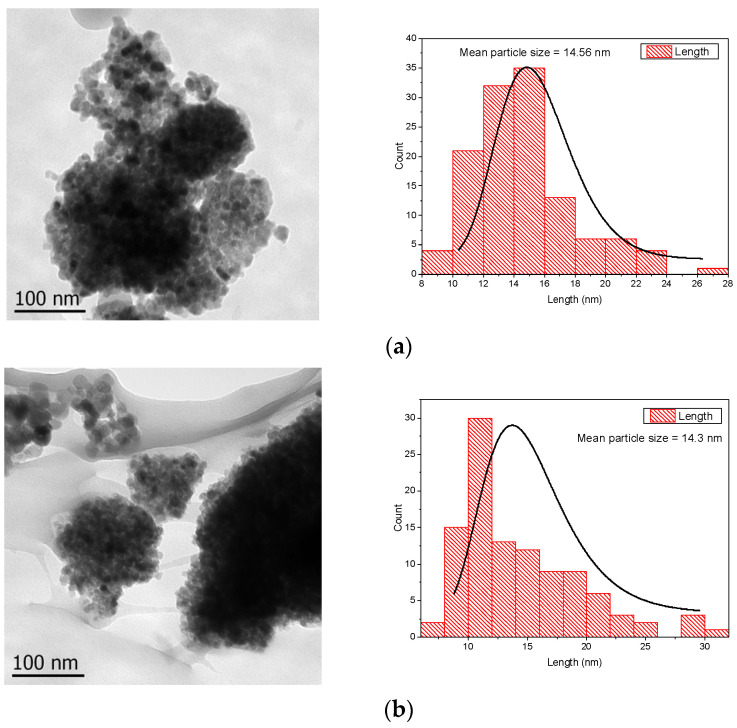

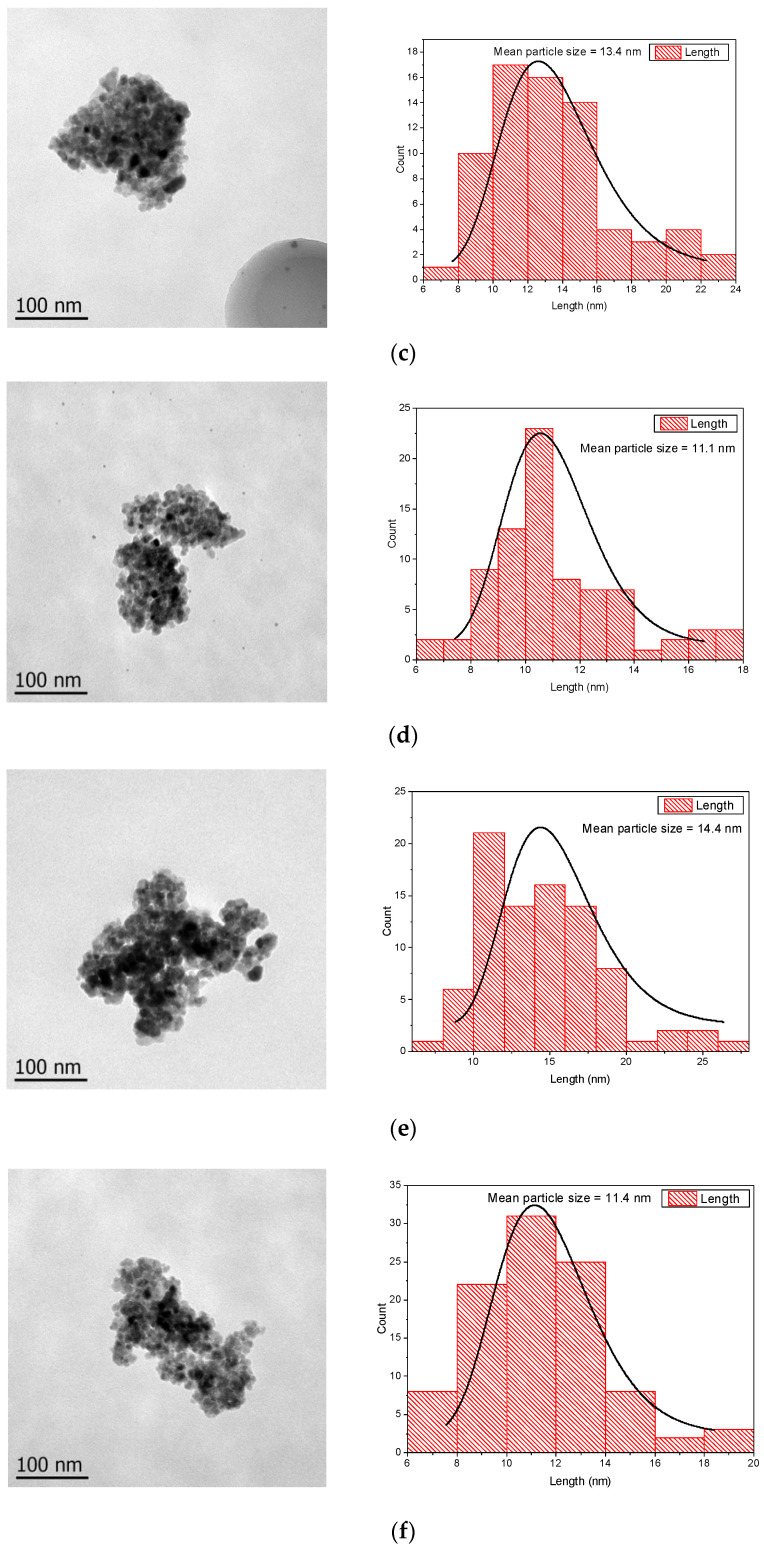
TEM micrographs and NP size distributions of (**a**) TiO_2_ SG, (**b**) TiO_2_ MW, (**c**) NaBH_4_/Ag-TiO_2_ SG, (**d**) NaBH_4_/Ag-TiO_2_ MW, (**e**) plant extract/Ag-TiO_2_ SG, (**f**) plant extract/Ag-TiO_2_ MW.

TEM analysis made it possible to approximate the NPs’ size according to the synthesis method and reductive reagent. In Figure 5a,b, corresponding to samples prepared by SG and MW, it is observed that with the use of MW in the TiO_2_ synthesis a slight change in size was presented, from 14.56 nm to 14.3 nm, and almost-round particles are noticed in both materials.

For the Ag-TiO_2_ materials using the NaBH_4_ reagent, the particle size changes from 13.4 nm to 11.1 nm when the synthesis method is coupled with MW (Figure 5c,d). Similar results are observed in the plant extract/Ag-TiO_2_ nanomaterials (Figure 5e,f). When the synthesis method is assisted with MW, the particle size is around 11.4 nm, and by using the sol-gel method, the size is 14.4 nm. Moreover, it can be noticed that the reductive reagent does not affect the NP size, only the synthesis method. Furthermore, by comparing with the un-doped material, with the presence of Ag in the MW-assisted sol–gel method the NP size decreases, despite the reduction reagent.

#### 3.2.3. Cristalographic Analysis (XRD and Raman Spectroscopy)

**Figure 6:**
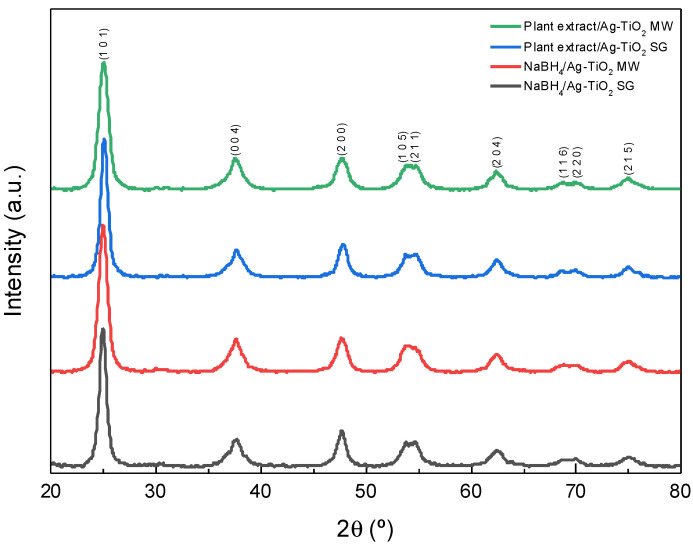
Ag-TiO_2_ X-ray diffraction patterns (**-**) NaBH_4_/Ag-TiO_2_ SG, (**-**) NaBH_4_/Ag-TiO_2_ MW, (**-**) plant extract/Ag-TiO_2_ SG, (**-**) plant extract/Ag-TiO_2_ MW.

Figure 6 shows the X-ray diffraction patterns of the silver-doped titanium dioxide materials prepared under different conditions. All the patterns reveal that crystalline materials were obtained. Nine Bragg diffraction lines at 2*θ* = 25.01°, 37.62°, 47.69°, 53.80°, 54.69°, 62.51°, 68.97°, 70.02°, and 74.96°, characteristics of the anatase phase of TiO_2_ (JCPDS 21-1272), are observed [35,37,38,50,82]. The metallic silver signals are not detected, which may be attributed to the low amount of silver nitrate used in the synthesis method.

The Scherrer equation determined the crystallite size, $D=\frac{K\lambda }{d\cos\theta }$, where *D* is the crystallite size, *K* is the crystallite-shape factor (0.9 is a good approximation), λ is the wavelength of the X-rays (1.5406 Å, Cu Kα), *d* is the full width at half the maximum of the diffraction peak in radians, and *θ* is the Bragg angle. The obtained results are presented in Table 3, where it is observed that the MW coupled with the synthesis method represents a change in the crystallite size as expected, but this is not by the chemical or bio-reductive agent presence; the crystallite size decreases as the NP size decreases.

Applying the Williamson–Hall method to determine the crystallite size [84,85], it is possible to obtain a better approximation of the crystallite size. This method considers the stress to which the network may be subjected, especially when the material could be doped.

**Figure 7:**
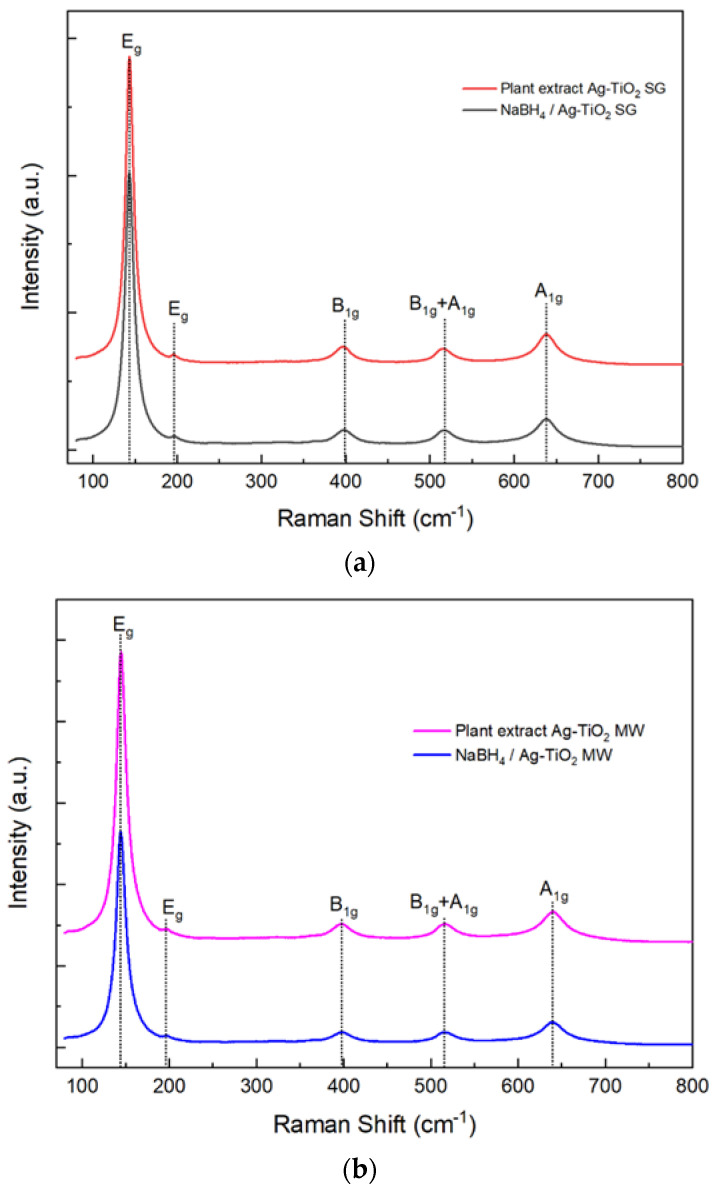
Ag-TiO_2_ Raman spectra were synthesized by: (**a**) the sol–gel method and (**b**) the MW-assisted sol–gel method.

Figure 7a,b shows the Raman spectra of the samples prepared using the sol–gel and MW-assisted sol–gel methods. The four spectra present vibrational features at 143.4 (E_g_), 195 (E_g_), 397.5 (B_1g_), 515.9 (B_1g_ + A_1g_), and 639.3 (A_1g_) cm^−1^, corresponding to the anatase phase of titanium dioxide.

**Figure 8:**
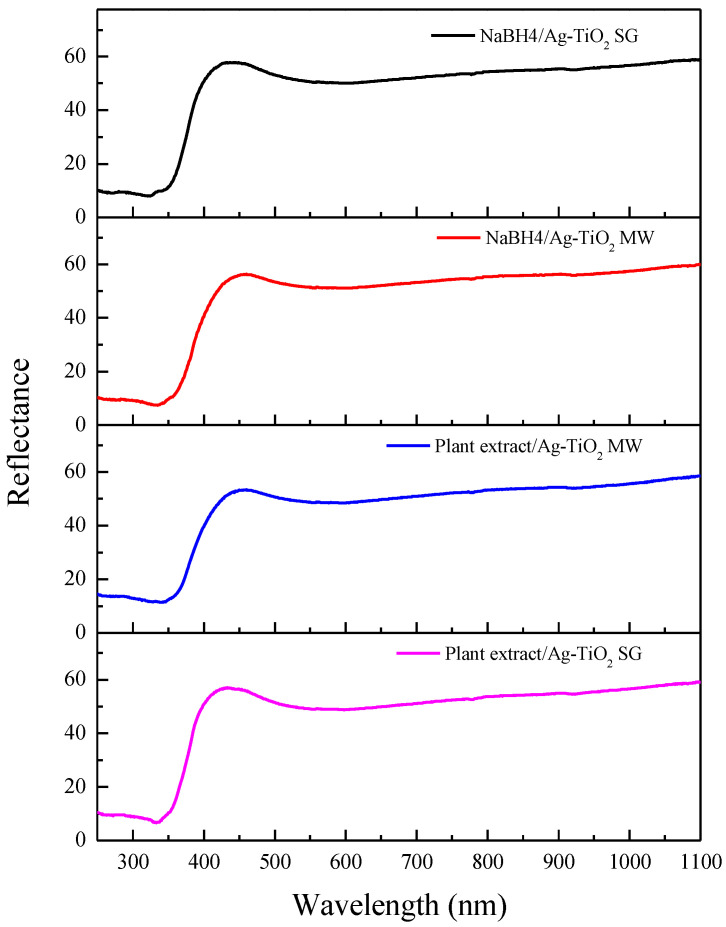
Ag-TiO_2_ DRS spectra (**-**) NaBH_4_/Ag-TiO_2_ SG, (***-***) NaBH_4_/Ag-TiO_2_ MW, (***-***) plant extract/Ag-TiO_2_ SG, (***-***) plant extract/Ag-TiO_2_ MW.

**Figure 9:**
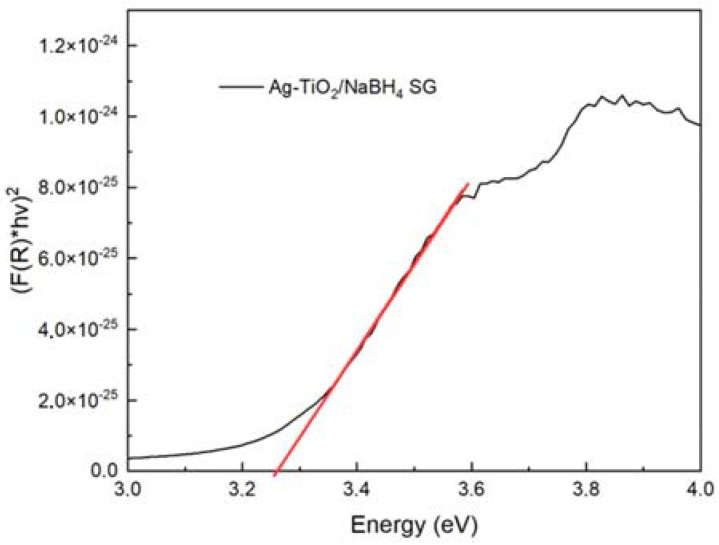
Tauc plot of NaBH_4_/Ag-TiO_2_ SG material.

The UV–Vis diffuse reflectance spectra (DRS) of the different samples are shown in Figure 8. These spectra show an absorption edge in the UV region (l < 425 nm), in agreement with the bandgap of TiO_2_, as expected. The absorption edge shifts to the visible spectral region when the MW-assisted process is employed for the synthesis; no effects due to silver incorporation into the TiO_2_ are observed. The corresponding optical bandgaps (Eg) were determined using the Tauc method; this was conducted by transforming the reflectance spectra to the Kubelka–Munk function, F(R), and then plotting (F(R)∙E)^2^ versus E [64]. The bandgap values were obtained by a linear fit of the linear portion of the curve, determining its intersection with the photon energy axis. Figure 9 shows an example of the (F(R)∙E)^2^ versus E graphs as well as the corresponding linear fit. In general, good fits to the different curves were obtained for the different samples.

The bandgap values of the materials prepared by both methods are shown in Table 4. These values agree well with the bandgap of the anatase phase, and no modification of the bandgap values was found due to the silver incorporation [61].

#### 3.2.4. XPS

**Figure 10:**
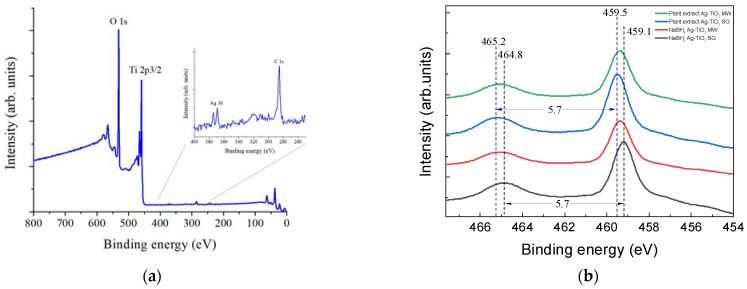

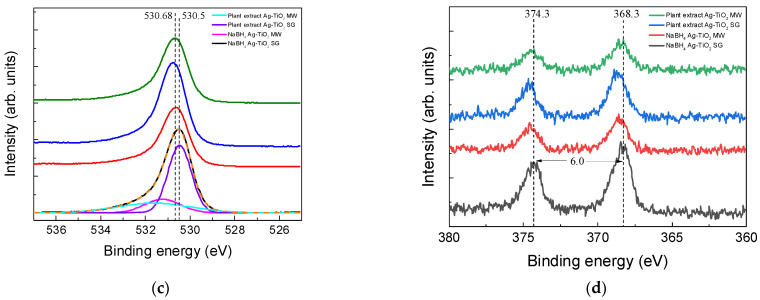
XPS spectra of Ag-TiO_2_ materials: (**a**) survey, (**b**) titanium, (**c**) oxygen, and (**d**) silver.

X-ray photoelectron spectroscopy (XPS) was employed to gain insight into the interaction of the Ag with the TiO_2_. Figure 10a shows the survey spectrum of one of the samples; two intense signals corresponding to O and Ti appear, confirming the presence of TiO_2_. The inset shows a zoom from 400 to 250 eV, revealing the presence of Ag and C with very low intensity. The carbon signal is attributed to adventitious carbon adsorbed from the environment. It is worth mentioning that all the samples showed almost identical XPS survey spectra. Figure 10b shows the Ti 2p region’s high-resolution XPS spectra for the different samples. Two peaks around 459.1 and 464.8 eV corresponding to the Ti 2p^3/2^ and Ti 2p^1/2^ orbitals, respectively, with a difference of 5.7 eV between them, are observed. These agree well with titanium as Ti^4+^ indicates the formation of TiO_2_ [86]. Slight shifts in binding energy depending on the preparation method suggest minor modifications in their chemical states.

Figure 10c displays the high-resolution XPS spectra for the O-1s region. These spectra show asymmetric peaks with tails extending to high binding energies. These peaks were fitted using the Voigt line shapes shown for the sample Ag-TiO_2_ prepared by the SG method using the NaBH_4_ reagent. The most intense peak, around 530.5 eV, is attributed to the O–Ti bonds, indicating the presence of TiO_2_ [87]. The peak close to 531.2 and 531.6 eV is attributed to O-H bindings, which are due to adsorbed moisture from the atmosphere [87]. Again, slight shifts in binding energy, depending on the preparation method, suggest that minor modifications in their chemical states are observed.

Figure 10d shows the high-resolution XPS spectra for the Ag 3d region. Two peaks, respectively, are observed around 368.3 and 374.3 eV and are attributed to Ag 3d^5/2^ and Ag 3d^3/2^ orbitals. The Ag 3d spin-orbit split of 6.0 eV [88] confirms the presence of metallic silver in the prepared samples.

### 3.3. Antimicrobial Activity

Silver nanoparticles were expected to have the highest percentage of inhibition, in agreement with all the existing evidence of their antimicrobial activity [89]. The difference between the inhibitory percentage obtained between the conventionally synthesized Ag NPs and the biosynthesized ones can be attributed to the eucalyptus extract functioning as a stabilizing agent, preventing the NP agglomeration and allowing the release of Ag^+^ ions [90]. The results obtained for the antimicrobial activity are presented in Table 5.

**Figure 11:**
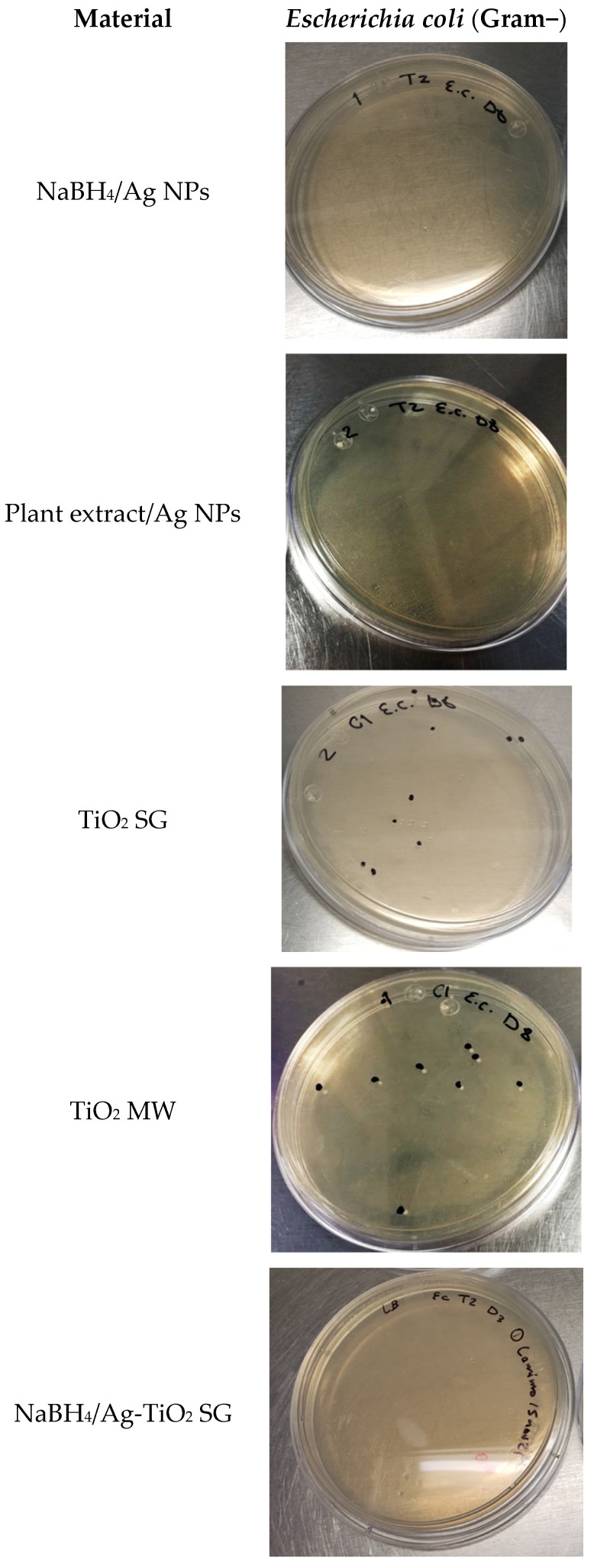

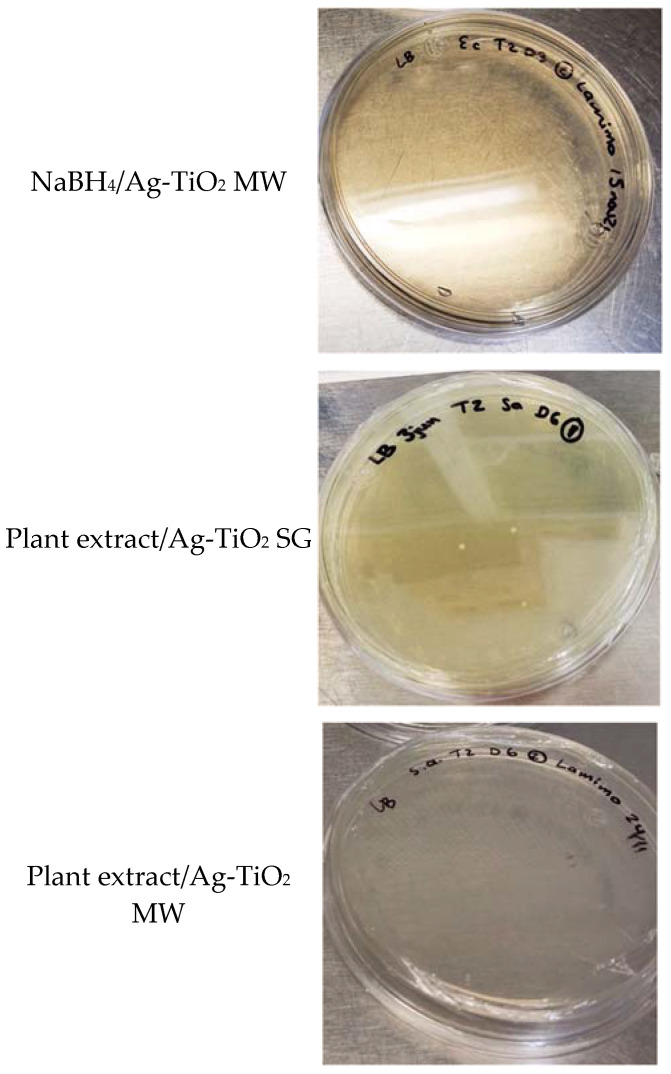
Images of bacterial inhibition growth of Ag NPs, TiO_2_, and Ag-TiO_2_ materials.

For Gram-positive and Gram-negative bacteria, it has been reported that the effect of Ag NPs is more significant for Gram-negative bacteria because of their thinner cell wall (3–4 nm), compared to 30 nm for Gram-positive bacteria [91]. Undoped titanium dioxide shows low inhibition percentages because the experiments were carried out without providing a UV light source that would allow the material to be activated. Representative images are shown in Figure 11, corresponding to the silver NPs, TiO_2_, and Ag-TiO_2_ materials against *Escherichia coli* growth.

Regarding the Ag-TiO_2_ materials synthesized with the conventional reductive reagent, it is possible to achieve high inhibition percentages (greater than 99%), which are directly attributed to the antibacterial silver properties. The materials synthesized with the *Eucalyptus globulus* L. plant extract showed high inhibition percentages, reaching 100% when the synthesis method is assisted with microwaves. These results are comparable to those obtained by the NaBH_4_/Ag-TiO_2_ MW materials. They suggest that the NP and crystallite size play an essential role in the bacteria–materials contact, producing damage to the cell membrane and slowing down their reproduction [92].

## 4. Discussion

The *Eucalyptus globulus* L. extract obtained by using ethanol as a solvent proved to be efficient in reducing the silver nitrate to silver NPs, with regard to the metabolites evaluated in this work. According to Barzinjy et al. (2020), and related to the UV–Vis spectra shown in Figure 1, the signal around 270 nm is in good agreement with the 250–270 nm regions, which refer to the electronic transitions of benzene and its products, which might comprise numerous aromatic compounds, such as phenolics, which are reached in the O–H groups [6], and flavonoids, through their OH group switching from the enol-mold to the keto-mold and donating a responsive hydrogen atom, which reduces the metallic ion into nanoparticles. Among those functional organic molecules, the flavonoid and phenol compound combinations existing in the extract are more likely to have had great empathies with the metal ion reductions [93]. The increment of the OH group, by using the mixture of alcohol/water, is expected to be observed in the absorbance signal increase.

For the FT-IR spectra (Figure 2), the wide absorbance band at 3336 cm^−1^ is related to the stretching vibration of alkynes, alcohols, and phenols, and it increases as the water is added to the solvent mixture. The overlapping signals between 2974 and 2850 cm^−1^ can be assigned to the C–H stretching vibrations in the CH/CH_2_/CH_3_ groups. The peak observed at 1653 cm^−1^ may be attributed to the N–H bending vibrations of alkenes, primary amines, and amides. The peak at 1088 cm^−1^ might be related to the stretching vibration of the C–O bond from the alcohols, esters, carboxylic acid, or ether. Finally, the signal at 1045 cm^−1^ is typical for the C–N stretching vibration of amines in proteins. These results were similarly obtained by Balčiūnaitienė et al. (2022), using a mixture of ethanol/water as a solvent [94].

The UV–Vis and FT-IR spectroscopies show that the phenolic compounds are the main extracted elements and might play an essential role in synthesizing metallic nanoparticles via green reduction. The secondary metabolite evaluation (Table 2) allowed the confirmation that the extract contained phenols and flavonoid compounds, with an adequate antioxidant activity by the DPPH and ABTS assays in comparison with the report by Palma et al. (2021) with similar extraction conditions [72]. The ethanolic extract showed a higher phenol content than for the rest of the treatment and a similar antioxidant activity by ABTS, except for its water mixture, with regard to the flavonoids content; the DPPH antioxidant activity is not the lowest, showing its reductive potential to form the silver nanoparticles, as can be observed in Figure 3 [5,95].

The synthesis methods applied were in good agreement with those previously reported by our research group, showing that the biosynthesis can be coupled to the sol–gel and microwave-assisted sol–gel method without a substantial morphology change, as seen in Figure 4 and Figure 5. As expected, the formation of the anatase phase was obtained due to the low silver concentration added to the synthesis and the thermal treatment (450 °C); no other crystalline phase was observed (Figure 6), and it was confirmed by the Raman spectra (Figure 7). Nevertheless, the bandgap was expected to be reduced by adding the silver to the TiO_2_ matrix. However, it was not the case for any of the materials, showing that the silver NPs are only over the surface, as confirmed by the XPS analysis (Figure 10). Similar reports have not been found using the *Eucalyptus globulus* L. extract to reduce silver NPs over TiO_2_. Nevertheless, some papers using the same plant extract to synthesize TiO_2_ show similar results regarding the morphology, crystallinity, and catalytic activity [58,96]. At this point, no remarkable changes in the physicochemical properties of the Ag-TiO_2_ materials obtained by the conventional or the biosynthesis methods are observed, leading to the assumption that the NaBH_4_ replacement as a reductive agent is feasible.

In terms of the affinity of the materials for Gram-positive and Gram-negative bacteria, in a general way and by the presented results, it can be said that the obtained materials are considered adequate and non-specific for inhibiting Gram-positive or Gram-negative bacteria. The primary mechanism that Ag NPs follow to inhibit bacteria is still not completely clear because it does not attack them through a single pathway. However, microorganisms do not seem to acquire resistance against silver [97]. The antimicrobial action of Ag NPs is linked to four well-defined mechanisms: (1) the adhesion of the nanoparticles to the surface of the cell membrane and wall; (2) the penetration of the NPs into the cell-damaging intracellular structures and biomolecules; (3) the NP-induced cellular toxicity and oxidative stress caused by the reactive oxygen species (ROS) and free radicals produced; and (4) the modulation of the signaling pathways related to cell transduction [98]. Either mechanism works for the application of these materials to water disinfection processes. The ability to inhibit the growth of *E. coli* (Gram-negative) encourages the carrying out of more studies related to clean wastewater, considering the high population of enterobacteria present, such as species of *Salmonella*, *Shigella,* or *Vibrio* [62]. The silver NPs are on the TiO_2_ surface, as seen in the XPS analysis and by the non-bandgap modification. Nevertheless, a stability test must be carried out for further experiments in water disinfection to assure a reasonable lifetime of the Ag-TiO_2_ materials.

## 5. Conclusions

It was possible to establish the proper extraction solvents to obtain the *Eucalyptus globulus* L. plant extract with adequate reductive compounds for the biosynthesis of silver NPs. According to the results observed in the UV–Visible and FT-IR spectroscopies, the different solvents and mixtures were adequate to extract the –OH compounds related to phytochemical constituents with phenols, amines, carboxyl, and carbonyl functional groups, as confirmed by the analysis of the secondary metabolites. The total phenol content obtained by the ethanol marked the difference in the silver nanoparticles formation. The obtained morphology of the materials differs between non-microwave-assisted synthesis and microwave-assisted synthesis, as was expected in comparison to our research group’s previous reports [61,62,63], and no further morphology changes were observed regarding the biosynthesis application to reduce the silver ions over the TiO_2_.

The shape and size of the TiO_2_ nanoparticles were evaluated by TEM, obtaining spheres in most cases and particle agglomerates with a size range of 11–14 nm. In terms of crystallinity, X-ray diffraction and Raman spectroscopy analyses confirmed that the titanium dioxide is in the anatase phase, and the silver added to the TiO_2_ did not produce materials with a narrow bandgap or crystalline phase changes. As the antibacterial activity experiments were performed without any light source, the results suggest that the bacteria inhibition growth was due to the presence of silver over the titanium dioxide surface, as observed in the obtained XPS analysis.

The bacteria inhibition values obtained for the biosynthesized material are promising for its possible application in systems where bacteria are a concern, such as the disinfection of biologically treated water or water potabilization. The similarity between the results obtained for conventional synthesis and biosynthesis allows us to propose the substitution of toxic chemicals such as sodium borohydride by plant extracts without sacrificing the properties of the material and to open the application of already existing materials to fields that involve direct contact with living beings, eliminating purification steps, and eliminating traces of toxic agents in the synthesis processes.

## Figures and Tables

**Table 1:** Volume ratios for the antimicrobial activity tests.

	Control (μL)	Treatment (μL)
LB medium	225	75
Bacteria	225	225
Material	0	150
Total volume	450	450

**Table 2:** Secondary metabolite evaluation in the *Eucalyptus globulus* L. extracts.

Solvent	Total Phenols Content (GAE mg/sample g) ^1^	Flavonoids Content (RE mg/sample g) ^2^	Antioxidant Activity (DPPH) (% Inhibition)	Antioxidant Activity (ABTS) (% Inhibition)
EtOH	58 ± 1 ^A^	172 ± 12 ^C^	86.63 ± 0.02 ^B^	98.690 ± 0.002 ^A^
MeOH	46 ± 7 ^AB^	299 ± 27 ^A^	82.31 ± 0.02 ^C^	99.830 ± 0.001 ^A^
EtOH/H_2_O	54 ± 15 ^A^	217 ± 11 ^B^	94.18 ± 0.01 ^A^	76.60 ± 0.01 ^B^
MeOH/H_2_O	45 ± 2 ^B^	161 ± 15 ^C^	94.80 ± 0.01 ^A^	98.990 ± 0.002 ^A^

^1^ mg GAE/sample g (mg Gallic Acid Equivalents/sample g), ^2^ mg RE/sample g (Rutine Equivalents/sample g). The average represents the value of 3 repetitions. Comparison between means (Tukey α ≤ 0.05). Means with different letters in the same column are statistically different.

**Table 3:** Ag-TiO_2_ Crystallite size.

Material	Debye–Scherrer (nm)		Williamson–Hall (nm)	
	SG	MW	SG	MW
TiO_2_ [38,83]	20.0	12.3	---	9.5
NaBH_4_/Ag-TiO_2_	8.3	7.6	7.8	6.4
Plant extract/Ag-TiO_2_	7.9	7.4	6.5	5.9

**Table 4:** Ag-TiO_2_ bandgap values.

Material	Bandgap (eV)	
	SG	MW
TiO_2_ [61]	3.2	3.1
NaBH_4_/Ag-TiO_2_	3.2	3.1
Plant extract/Ag-TiO_2_	3.2	3.1

**Table 5:** Ag-TiO_2_ bacterial inhibition growth percentages.

Material	Inhibition %	
	*Staphylococcus aureus* (Gram+)	*Escherichia coli* (Gram−)
NaBH_4_/Ag NPs	85 ± 8	Total
Plant extract/Ag NPs	Total	Total
TiO_2_ SG	Undetermined	30 ± 7
TiO_2_ MW	66 ± 4	17 ± 7
NaBH_4_/Ag-TiO_2_ SG	99.82 ± 0.01	Total
NaBH_4_/Ag-TiO_2_ MW	Total	Total
Plant extract/Ag-TiO_2_ SG	99.1 ± 0.6	96 ± 3
Plant extract/Ag-TiO_2_ MW	Total	Total

## Data Availability

Not applicable.
